# Non-linear model predictive control for variable speed hydropower

**DOI:** 10.1038/s41598-025-28361-3

**Published:** 2025-12-24

**Authors:** Tajana Nepal, Tommy Sneltvedt, Choukha Ram, Diwakar Bista, Thomas Øyvang, Roshan Sharma

**Affiliations:** 1 Department of Electrical Engineering, IT and Cybernetics, University of South Eastern Norway, Porsgrunn, 3917 Norway; 2 Department of Electrical and Electronics Engineering, Kathmandu University, Dhulikhel, 45210 Nepal

**Keywords:** Ancillary services, Coordinated control, Control vector, Model based control, Prediction horizon, Virtual inertia, States, Energy science and technology, Engineering

## Abstract

Inverter Based Resources (IBR) are gaining more popularity in modern power systems, leading to decrease in grid inertia. Hydropower Plants with rotating synchronous machines have been excellent source of inertia in existing grids. Adding a frequency converter between the generator and the grid in these hydropower plant would enable to run the hydropower in variable speed leading to enhanced efficiency and more flexibility in operation. These Variable Speed Hydropower Plant (VSHP), having the capability of anciliary services for grid support could be a potential solution for providing synthetic inertia to low-inertia power grids. However, there is a little research done on control of VSHP for grid support. This paper aims at implementing Non-Linear Model Predictive Controller (NLMPC) algorithm for optimal control of VSHPs, and the results are compared with classical PID control method. Explicit comparison under realistic operating condition and analysis of constraint handling capabilities and robustness of MPC have been performed. Advanced model based NLMPC successfully coordinated the hydraulic and electric systems having different time constants by implementing multi-objective optimization and upstream constraints satisfaction. Furthermore, the NLMPC has shown relatively better performance than the classical controllers even during the occurrence of an unanticipated grid-side disturbance. This paper is claimed to be an important work in control domain since it develops the control system for VSHPs using time-domain models which can offer better insights over the nonlinearities that exist within hydropower systems. Apart from the computational complexity, NLMPC is found to be viable for the control of VSHP since it ensured stable operation, as can be seen from the analysis of different operational cases studied implemented in this paper. The key takeaway from this paper is the potential of utilizing advanced model based optimal control strategy for coordination among complex dynamic systems of VSHP for different loading patters and disturbances. Also, the MPC has a scope of providing more robust grid support by addressing overloads and uncertainties during operation.

## Introduction

By 2025, renewable sources will surpass coal as an electricity production source, representing up to 35% of global electricity generation^1^. As the shift toward inverter-based resources (IBRs) continues, there are increasingly more Power Electronic Converters (PECs) on the grid, and fewer conventional rotating machines. Thus, grid operators worldwide are faced with new challenges in maintaining system stability. One of the emerging solutions is the creation of specialized markets that reward power plants for quickly ramping up or down their output to stabilize grid frequency, a technique referred to as Fast Frequency Response (FFR). Traditionally, hydropower power plants have played a leading role in grid stability, with better voltage and frequency control^2^.

Variable Speed Hydropower Plants (VSHPs) are proving to be a potential choice to address the challenge of high renewable integration^3^. VSHPs can modulate turbine speed to change output quickly, with faster active power control compared to traditional fixed-speed hydropower power plants. The advantages of VSHP technology may be highlighted in three major points

- Increased efficiency: Incentivizing turbine speed at partial loads can result in an increase in efficiency of 3–10%,
- Greater operating range: VSHPs are able to operate at a wider head ratio, withstanding large water level fluctuations, and
- Reduction of mechanical stresses: Lower vibration and cavitation levels reduce maintenance loads and extend equipment life^4^.

Along with such mechanical benefits, VSHPs equipped with converter technology can offer ancillary services through the concept of Virtual Inertia (VI). These include better frequency regulation, voltage and reactive power support, and overall system stability facilitating higher penetration of IBRs into the grid^5^. Such a prospect is particularly relevant in the aftermath of events like the recent Spanish black out that happened in spring of 2025, where nearly 70% of the electricity was of renewable origin^6^. Although the underlying reason is unclear, the lack of built-in inertia in renewables is also suspected to be responsible. In that regard, VSHPs with robust control schemes can perform the role of a stabilizing force, mitigating low-inertia risks.

Furthermore, VSHPs allow for flexibility in addressing renewable intermittency, enabling minimization of power variation and curtailment avoidance. VSHP integration also enables more efficient use of available transmission infrastructure^7^. However, these benefits come at the cost of increased complexity in the system, which requires coordinated control of hydraulic dynamics, turbine-generator efficiency, power electronics, and grid interaction.

While the global energy transition is picking up pace, cost-effectiveness becomes a priority concern. Lan et al.’s research shows that while VSHPs carry higher upfront investment than fixed-speed plants, their value increases as higher penetration of renewables is achieved^8^. It determines that there exist numerous factors which are crucial for economic viability. Some of them are: (a) Economic frameworks that appreciate and reward VSHPs for offering ancillary services, (b) Strategic synergy with variable renewables to counter variability, and (c) Design of control techniques optimized to achieve maximum performance over varying operating conditions.

This paper tries to address the last factor which demands for advanced control strategies indicating the inadequacy of classical PID controllers, which in their traditional form may not be able to deliver optimal performance. To counter this, in this paper we propose a coordinated nonlinear MPC controller to regulate hydraulic and electrical subsystems as a multi-objective optimization problem. It is simulated under various VSHP operating conditions and compared with PID controllers to demonstrate its effectiveness and superiority in optimally controlling the VSHP.

### Literature review

Numerous control methods have been employed in hydropower plants to enhance their flexibility and performance. Some of these include methods such as Neural Networks^4^, conventional PID controllers coupled with advanced control strategies^9^, two-sequence PI controllers for effective governor and droop control^10^, and small-scale control strategies such as Direct Power Control and Maximum Power Point Tracking (MPPT)^11^. In addition, fuzzy logic configurations have been employed to track best operating points and maximize power extraction^12^. These methods have been the baseline for control since long time, however they leave a room for improvement because of their drawbacks like poor adaptability, lack of constraint handling capacity, inadquete for multi-varaible systems, etc.

As a better option, Model Predictive Control (MPC) has emerged as a modern tool in recent years to provide effective local and centralized control configurations for hydropower systems. For instance, authors employ a local MPC configuration in a conventional hydropower plant against hydro turbine governor control employing linear models of the turbine and generator^13^. On the contrary, centralized MPC-based Load Frequency Control (LFC) techniques have been proposed by Beus et al and Nagode et al in their work, where grid-side factors such as tie-line flows, active power load variations, and transmission lengths are considered for frequency regulation^14,15^. But this linear MPC assumes linear dynamics and its inability to capture and handle non-linear dynamics and constraints makes it limited for application in linearized systems only. Also, for grid side control, authors present a control method based on Cascaded Non -Integral Second order Integrator(CNISOGI) and Repetitive Controller(RC) in their work, which has made a significant contribution on harmonics suppression with smooth bidirectional energy flow^16^. This is a robust control method for grid interactive non-linear loads. However, when we need to work together on systems like hydropower, involving entirely different dynamics with significantly different time constants (in the order of milliseconds and minutes), a control method capable of handling both the dynamics at a time is needed.

Under the renewable integration scenario, the work by Pandey and Winkler presents an MPC scheme for balancing fluctuating solar and wind generation with hydropower in a microgrid environment^17^. This decentralized control approach regulates multiple sources of energy optimally. On similar grounds, authors suggest real-time MPC for Run-of-River (RoR) power stations using a hybrid data-driven and physics-driven model^18^. This new generation control algorithm surpasses traditional MPC by predicting the state more effectively, correcting through feedback, and having a reduced decision space. While hybrid data-driven models have huge horizon to offer in the field of control, they are highly dependent of data quality and quantity leading to questions on interpretability of the model sometimes. The extension of MPC to VSHPs further adds flexibility to hydropower operation. Most of the existing work considers pumped-storage VSHPs with pump turbines having variable power consumption during pumping mode. They provide energy storage and load regulation, and hence are a valuable tool for flexible plant operation and grid support^18,23,24^. Pump storage plants are very good application of VSHPs since they have flexibility of power consumption in pumping mode, however the ones using synchronous generators with full size converters and suitable control system could contribute more in grid support.

The authors present a VSHP model based on a converter-fed synchronous machine (CFSM) which contains a hydraulic turbine, permanent magnet synchronous generator, and power electronic converter^19^. The model is utilized to simulate two parallel hydropower units under identical hydrological conditions, and an experimental proof is provided for a three-layer multi-objective control strategy based on an advanced perturb-and-observe algorithm. Simulating parallel hydropower units and implementing multi-objective control makes this research look more similar to the real systems. This control strategy could be made more applicable by involving a multi-objective optimization which could fulfill multiple objectives simultaneously handling system constraints.

Further developments are seen in Reigstad and his co-author’s work, where VSHP models for grid integration studies are presented, including virtual inertia control schemes^5,20^. Building on this, the same authors present a linear MPC that makes it possible to use Fast Frequency Reserves (FFR) while considering hydraulic and electrical constraints^21^. The controller adjusts the guide vane opening of the turbine and, while satisfying hydraulic constraints the power output reference of the VSHP is adjusted. The approach is extended further where an NLMPC is used with the a more accurate nonlinear model of the power plant^22^. The controller reduces power oscillations to the grid, decreases water hammer impacts to the penstock, and coordinated turbine controller with Virtual Synchronous Generator (VSG) control.

A brief summary of the major contributions from existing methods is shown in Table 1, which helps us highlight the research gap. Existing research is mostly limited in terms of linearized models that lack the ability to capture detailed nonlinear dynamics of the system. Time-domain models are better suited for real-time control and analysis when system nonlinearities play a major role.

**Table 1:** Comparative summary of existing control strategies in hydropower systems and proposed work in this paper.

Method	Description	Work done
Neural Networks^4^	Nonlinear modeling and control of hydropower with data driven learning.	Employs purely data based learning of nonlinear dynamics without explicit physical modeling.
Conventional PID and hybrid PID based control^9^	Governor and grid frequency regulation by empirical adjustment.	Based on Ziegler Nichols or empirical performance; purely feedback based with no prediction or optimization.
Fuzzy logic and MPPT based control^11,12^	Optimal power tracking and efficiency maximization.	Introduces heuristic, rule based decision logic for nonlinear systems.
Local linear MPC for hydro turbine control^13^	Linear MPC for turbine generator control.	Uses linearized state space model for short horizon predictive control.
Centralized MPC for Load Frequency Control (LFC)^14,15^	Grid frequency and tie line power coordination.	Centralized predictive optimization using linear grid model with constraints.
Decentralized MPC for hybrid microgrids^17^	Coordination of multiple renewable sources.	Decentralized optimization based local MPC controllers; reduces dependency on communication.
Hybrid data driven and physics driven MPC^18^	Run of River (RoR) real time MPC.	Blends data driven learning with the physics based equations employed for state estimation.
VSHP model with converter fed synchronous machine (CFSM)^19^	Converter fed VSHP with multi-objective perturb and observe control.	Merges experimental and simulation based verification; partial multi-objective coordination.
Linear and Nonlinear MPC for VSHPs^5,20–22^	Frequency regulation and inertia emulation in VSHPs.	Linear MPC for FFR; nonlinear MPC includes plant dynamics and constraint handling; Modeling and control in s-domain.
Proposed Nonlinear Multi-objective MPC (This work)	Converter fed VSHP power and frequency control.	Integrated nonlinear MPC with multi-objective optimization and explicit constraint management, considering turbine, generator, converter dynamics; Modeling and control performed on t-domain.

### Research gap

While previous studies have worked on control of Doubly-Fed Induction Generators (DFIG) for pump storage plants^24^, and on synchronous generators for VSHPs^19^, there is a need of extensive research on advanced control strategy of VSHPs for grid support. To the best of our knowledge, no previous research exists on VSHPs using synchronous generators with time-domain models. Even though most of the aforementioned models rely on s-domain modeling and inverse Laplace transformations which are linear in nature, time-domain modeling can offer better insights over the nonlinearities that exist within hydropower systems. Also, the control implementations have either focused on enhancing turbine efficiency while satisfying the constraints on hydraulic side, or only looked at grid side with more priority for frequency support while making simplified assumptions on the upstream dynamics and parametric variations. A controller capable of working together with slow-acting water dynamics and fast-acting electrical dynamics, and which is based on a non-linear model of VSHP is lacking in literature.

Our previous work has proposed a full order non-linear dynamic model for a VSHP including generator, turbine and converter interactions^25^. The model can show major dynamics of VSHP and yet is simple enough to be used for the design of real time advanced controllers. In this paper, we attempt on developing a controller that can coordinate with both the hydraulic and the electric sides of the VSHP.

### Contribution of the paper

This article shows implementation of a NLMPC to a VSHP model proposed in^25^ and studies its performance for sudden load/disturbance variations. Unlike linear model-based MPC schemes, the present work makes full use of the non-linear mechanical and electrical subsystems that allow for improved performance against anticipated load changes as well as grid disturbances. Furthermore, this paper also proposes another research direction to alleviate grid overload by optimally sharing power among various generation units using advanced controllers. The key contributions in this paper are as follows:

- NLMPC controller design and its implementation to a VSHP based on time-domain modeling.
- Explicit comparison with the classical PID control under realistic operating constraints, and thus demonstrating the performance gap.
- Analysis of constraint handling capabilities, and dynamics performance with simulation under step, ramp and random load changes, impulse loads, and disturbances on the demand side.

This work has some practical interest, since the preferred NLMPC strategy offers a viable pathway for transforming legacy hydropower facilities into fleet, flexible, and grid-friendly resources for high renewable grids.

### Organization of the paper

The following sections in this paper chronologically present Methodology, Results, Superiority of MPC over PID, Discussion, and Conclusion. Methodology includes model summary, assumptions made in this work, and details of optimal control problem formulation. After methodology, the results section includes different simulation case studies results using NLMPC and PID control for VSHP operation. Superiority of MPC over PID section presents comparison showcasing superiority of MPC over classical control for improved operation of VSHP. Lastly, discussion on the results of various opertaional scenerios of the VSHP have been presented in detail, and some specific conclusion have been drawn to highlight the major contribution of this work in the last section.

## Methodology

This paper focuses on developing a NLMPC strategy for improved operation of VSHP. The mathematical model of the hydropower system that has been used as the prediction model for designing the model predictive control strategy in this paper is taken form our previous work^25^. This model is chosen for designing a model based controller since it can capture all the needed dynamics of the system and at the same time is simple enough to be used for real-time control and analysis.

Furthermore, the nonlinear MPC acts as a coordinating controller for controlling two different subsystems of variable speed hydropower plant. The first subsystem of VSHP is the hydraulic part that includes the entire water way from the reservoir to the turbine output. The second subsystem of VSHP is the electrical part that includes the generator, converter and the grid. These two subsystems have different time constants. The hydraulic part is much more slower than the electrical part in its response. It is challenging to design a coordinating controller for such systems having both very fast dynamics and slower dynamics. Its capacity to manipulate both the guide vanes at the hydraulic side and the reference power to VSG makes the nonlinear MPC particularly viable for the control of VSHP to enhance grid support possibilities.

### Model summary

**Fig. 1:**
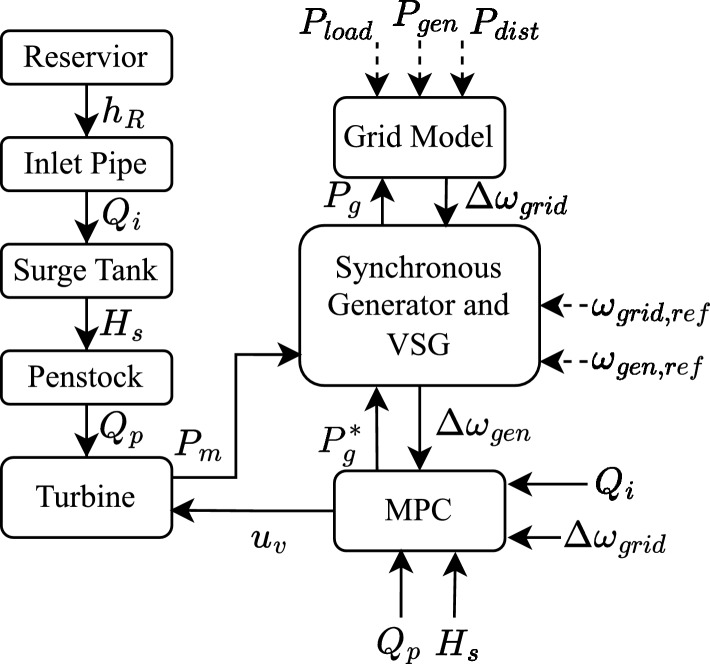
Functional diagram of VSHP showing all the states and control inputs.

As already stated in the previous section, the model used in this paper is presented in detail in our previous work. This section only briefly describes the equations in the model which involve major variables and the states for the sake of completeness. To make this more clear, a functional diagram showing different components of VSHP with all the variables, states and control inputs is shown in Fig. 1. From the figure, the major components of VSHP, namely: reservoir, inlet pipe, surge tank, penstock, turbine, generator, Virtual Synchronous Gneertaor (VSG) and grid are briefly explained and their mathematical model are shown in this section. For a more complete schematic of the VSHP showing its different components, please refer to our previous work^25^.

The reservoir is represented as a large pond and has no dominant dynamics to NLMPC since the level of water in the reservoir is considered to be constant. An inlet pipe connecting the bottom of the reservoir to a surge tank with diameter $D_i$, length $L_i$, inclined height $H_i$ and with a rated inlet flow $Q_i$ is represented as in Equation 1. In the equation, $\rho$ represents the density of water, *g* represents the acceleration due to gravity, *f* represents the coefficient of friction, and $A_i$ represents the area of the pipe.

1 $$\begin{aligned} \displaystyle \frac{dQ_{i}}{dt} = \displaystyle \frac{A_{i}}{\rho L_{i}} ( P_{R2} -P_{s,1}) - \displaystyle \frac{ \pi D_{i} f }{8 A^2_{i}} Q_{i} | Q_{i}| + \displaystyle \frac{ g A_{i} H_{i}}{L_{i}} \end{aligned}$$

A cylindrical surge tank with water level $H_s$, surface area $A_s$, supplying water to a penstock with flow $Q_p$, and surge tank flow rate $Q_s$ may be represented mathematically as written in Eqs. 2 and 3. Similarly, a penstock with diameter $D_P$, cross sectional area $A_p$, and length $L_p$ with a flow rate of $Q_p$ is represented mathematically in Equations 4 and 5. In the equation of penstock, $P_t$ represents pressure in turbine, $C_v$ is a constant which gives information about turbine valve capacity and $u_v$ is the control input that denotes guide vanes opening.

2 $$\begin{aligned} \displaystyle \frac{dH_{s}}{dt}= & \frac{Q_{s}}{A_{s}} \end{aligned}$$

3 $$\begin{aligned} Q_{s}= & Q_{i} - Q_{P}\end{aligned}$$

4 $$\begin{aligned} \displaystyle \frac{dQ_{P}}{dt}= & \displaystyle \frac{A_{P}}{\rho L_{P}} ( P_{s,1} -P_t) - \displaystyle \frac{ \pi D_{P} f }{8 A^2_{P}} Q_{P} | Q_{P}| + \displaystyle \frac{ g A_{P} H_{P}}{L_{P}} \end{aligned}$$

5 $$P_{t} = P_{a} \left( {1 + \left( {\frac{{Q_{p} }}{{C_{v} u_{v} }}} \right)^{2} } \right)$$

A turbine generating mechanical power ($P_{shaft/mechanical}$) is represented mathematically using its inlet and outlet radii $R_1$ and $R_2$, inlet and outlet areas $A_1$ and $A_2$, speed of turbine-generator unit $\omega$, degree of guide vanes opening $\alpha _1$, and a turbine constant $\beta _1$. The mechanical power is also determined by the penstock flow $Q_p$ and rate of change of mass in turbine $\dot{m}$. A mathematical equation used to calculate the mechanical power generated by turbine may be written in Eqs. 6 and 7. More details about this turbine model can be found in lecture notes by Bernt Lie^26^.

6 $$\begin{aligned} P_{shaft/mechanical}= & \dot{m} \omega R_1 \left[ \displaystyle \frac{Q_p}{A_1} Cot\alpha _1 \right] - \dot{m} \omega R_2 \left[ \omega R_2 + \displaystyle \frac{Q_p}{A_2} Cot \beta _2\right] \end{aligned}$$

7 $$\begin{aligned} \dot{m}= & \rho * Q_p \end{aligned}$$

After the turbine, the electrical system is represented in per unit (PU). The base value of mechanical power $P_{base}$ is used to convert the mechanical power generated by the turbine to PU which is fed to the generator. The generator, VSG and the grid are modelled in PU. Swing equation using moment of inertia *H* of generator and the difference of input mechanical and output electrical power ($P_m-P_g$) to calculate rate of change of generator frequency $\omega _{gen}$ is used to model the synchronous generator. A damping coefficient *D* to represent oscillation damping is also kept in the equation to show its general purpose. Since this paper does not cover oscillation studies, *D* is assumed to be zero. The generator that deviates from synchronous speed is supposed to follow grid frequency $\omega _{grid}$ eventually. The change in generator frequency is represented by $\Delta \omega _{gen}$. The main mathematical equations used to represent the model of synchornous generator used in this paper may be written as seen in Eqs. 8, 9 and 10.

8 $$\begin{aligned} \omega _{gen}= & \Delta \omega _{gen} + \omega _{grid} \end{aligned}$$

9 $$\begin{aligned} \displaystyle \frac{d \Delta \omega _{gen}}{dt}= & \displaystyle \frac{1}{ 2 H \omega _{gen}}(P_m - P_g- D \Delta \omega _{gen}) \end{aligned}$$

10 $$\begin{aligned} P_m= & \displaystyle \frac{P_{shaft/mechanical}}{P_{base}} \end{aligned}$$

A Virtual Synchronous Generator (VSG), mathematically represented in Eq. 11 is the component that enables the VSHP to operate in synchronism with the grid by allowing the turbine-generator unit speed to vary as needed. The machine side converter, dc link capacitor and the grid side converter as a whole is modelled as a single equation with the main purpose of controlling active power injected to the grid $P_g$. The setpoint for this active power is $P_g^*$ which is optimally calculated by NLMPC algorithm. In the equation of VSG, $K_{VSG,p}$ symbolizes proportional time constant for VSG control loop and $K_{VSG,d}$ symbolizes derivative time constant for VSG control loop.

11 $$\begin{aligned} P_{g} = K_{VSG,p} \Delta \omega _{grid} + K_{VSG,d} \frac{d \Delta \omega _{grid}}{dt} + P_g^* \end{aligned}$$

The grid is modelled as large synchronous generator with moment of inertia $H_g$, current active power generation in the grid $P_{gen}$, and current active power demand in the grid $P_{load}$. This model also includes power imbalances or disturbances denoted by $P_{dist}$ and damping coffecient for the grid $D_m$. The grid as a whole is mathematically modelled by Eqs. 12 and 13. In the equation, $S_{n}$ denotes total rated power of all connected sources. For a grid injected by only one hydropower (the case of this paper), $P_{gen}$ is same as $P_g$. However, if there were multiple hydropower plants in the grid, $P_{gen}$ would be sum of $P_{g1}$, $P_{g2}$, $P_{g3}$, .. and so on.

12 $$\begin{aligned} \frac{d \Delta \omega _{grid}}{dt}= & \frac{\omega _{ref}}{2 H_g S_n} ( P_{gen} - P_{load} + P_{dist} - D_m \Delta \omega _{grid}) \end{aligned}$$

13 $$\begin{aligned} \omega _{grid}= & \omega _{ref} + \Delta \omega _{grid} \end{aligned}$$

### Assumptions

A nonlinear MPC is designed for controlling the entire system, both electrical and hydraulic part by managing the VSG setpoint for power generation and the control signal to the guide vanes respectively. Thus the variables to be optimized are: $u_v$= Control signal to guide vanes and $P_g^*$= Active power generation setpoint to VSG. A functional diagram showing all the variables, states and control inputs is shown in Fig. 1.

To speed up the computation time for solving the NLP problem, control input grouping/blocking has been performed. The vector of the decision variable supplied to the optimzer takes the form as seen in Eq. 14.

14 $$\begin{aligned} u_k = [u_{v1}, u_{v2}, \ldots ,u_{vN_p},p_{g1}^*, ,p_{g2}^*, \ldots , p_{g(N_p)}^*]^T \end{aligned}$$

here, $N_p$ is the Prediction horizon length. The deviation form of control signal may be written as: $\Delta u_{k} = u_k - u_{k-1}$. The main aim here is to maintain the grid frequency close to its setpoint which equals 1 per unit and maintain the generator frequency as close to the grid frequency as quickly as possible. The frequency of the grid might be disturbed due to load changes and disturbances which can be adjusted by manipulating the injected power to the grid which is done by changing the power setpoint to the converter. The generator frequency is allowed to deviate momentarily to lower values, but it is desired to catch up with grid frequency or its setpoint as soon as possible. This is done by changing the gates and opening the guide vanes. Thus, the error vectors can be stated as in Eqs. 15 and 16.

15 $$\begin{aligned} e_{grid}= & \omega _{grid,ref,k}-\omega _{grid} \end{aligned}$$

16 $$\begin{aligned} e_{gen}= & \omega _{gen,ref,k}-\omega _{gen} \end{aligned}$$

here, $\omega _{grid,ref,k}$ denotes setpoint for grid frequency, $\omega _{gen,ref,k}$ denotes setpoint for generator frequency, $\omega _{grid}$ denotes current grid frequency and $\omega _{gen}$ denotes current turbine-generator unit frequency. With MPC algorithm, these two errors will be minimized by solving an optimization problem. The objective function and the constraints of the optimal control problem are briefly described in the following sections. The error vector then takes the form in Eq. 17.

17 $$\begin{aligned} e_k = [e_{grid_1}, e_{grid_2},... e_{grid_{N_p}},e_{gen_1}, e_{gen_2}, ... e_{gen_{N_p}}]^T \end{aligned}$$

### Objective function

Here, the objective function contains two criteria to meet, namely: minimize the error in grid frequency and make the generator follow the grid frequency, which is combined into one cost function by weights. This makes it a scalarized multi-objective optimization problem. The objective function for NLMPC used in this work is shown in Eq. 18.

18 $$\begin{aligned} J = \frac{1}{2} \sum _{k=1}^{N_p}(e_k^TQe_k + \Delta u_{k-1}^TH\Delta u_{k-1}) \end{aligned}$$

In this objective function, *Q* is the weighting matrix for error, *H* is the weighting matrix for control signal, which take the form as shown below. The values in the weighting matrices were manually tuned, based on the task we aim to prioritize. $Q_{grid}$, $Q_{gen}$, $H_{u_v}$, $H_{p_g^*}$ are diagonal square matrices with order *k* and having all the diagonal elements equal to 5000, 100, 100 and 0.1 respectively. Additionally, $e_k$ is the $k^{th}$ value of error, and $\Delta u_{k-1}$ is the $(k-1)^{th}$ value of variables to be optimized in deviation form. The prediction horizon and control horizon are equal which is taken to be 75 samples in this work.

$$Q = \begin{bmatrix} Q_{grid} & 0 \\ 0 & Q_{gen} \end{bmatrix} \quad H = \begin{bmatrix} H_{u_v} & 0 \\ 0 & H_{p_g^*} \end{bmatrix}$$

To calculate $e_k$ in the objective function given by Eq. 18, we need to solve the dynamic mathematical model of the VSHP for the whole prediction horizon length. The mathematical model of the system is given by a set of ordinary differential equations together with some algebraic equations as outlined in the section “model summary”. Thus the dynamic model of the system acts as the prediction model for the MPC algorithm to predict how the behavior of the system would change over the whole prediction horizon length.

### Constraints

#### Inequality constraints

The guide vane signal for the turbine cannot go above 100% and cannot be completely closed since this leads to numerical instability of the model. So, its opening is limited to from 5 to 100%. Also, the electrical power generated by the VSHP can range from 0 to 100%. So, the setpoint for VSG, which eventually is the setpoint for electrical power generated by VSHP varies from 0 to 1 pu. Bounds on decision variables are shown in Eq. 19.

19 $$\begin{aligned} \left\{ \begin{aligned}&0.05 \le u_{vk} \le 1 \\&0 \le P_{gk}^* \le 1 \end{aligned} \right\} \quad \forall \; k \in 1 \dots N_p \end{aligned}$$

Another set of constraints that should be applied is the limits on deviation in guide vanes opening. The deviation is mathematically given by $\Delta u_{vk} = u_{vk} - u_{v(k-1)}$. Since the rate of change of the guide vanes will affect the pressure in the upstream region and also might cause mechanical damage, this variable is limited. These constraints are stated in Eq. 20.

20 $$\begin{aligned} \left\{ \begin{aligned}&-0.1 \le \Delta u_{vk} \le 0.1 \end{aligned} \right\} \quad \forall \; k \in 1 \dots N_p \end{aligned}$$

Similarly, the upstream hydraulic flows and water levels also cannot violate their prespecified values. The surge tank water level is limited to stay between 10 meters and 40 meters which is the height of the surge tank. The lower value for water level also needs to be specified because if we allow the water level below this, the penstock might not receive full flow which might cause problems like injection of air bubbles leading to cavitation in turbine, the accumulated settled sand particles at the bottom of surge tank getting in the penstock pipe, decreasing water pressure in the penstock leading to water hammer, etc. Also, the penstock flow rate is limited between maximum of 16 m$^3/$s and 10% of its maximum value. These may be listed as inequality constraints as shown in Eq. 21.

21 $$\begin{aligned} \left\{ \begin{aligned}&H_{sk} \le 40, \quad H_{sk} \ge 10 \\&Q_{pk} \le 16, \quad Q_{pk} \ge 1.6 \end{aligned} \right\} \quad \forall \; k \in 1 \dots N_p \end{aligned}$$

The optimal control problem is formulated and solved in MATLAB(R2023B) script using *fmincon* solver with *SQP* algorithm in a 12th gen, 3.2 GHz PC with windows 11. The sampling time used was 0.01 sec with *TolFun* of $10^{-10}$ and maximum iterations limited to 5000. To solve the ordinary differential equations used to model the VSHP, Runge-Kutta fourth order integration is used.

### Classical governor control

PI based governor is used for classical control. The relaxed Ziegler Nichols PI tuning method is used to adjust the controller which gave $K_p$ = 62.5 and $K_i$ = 1.125. Unlike NLMPC, this controller is manipulating only the guide vales in the turbine, and not changing VSG setpoint. The VSG setpoint is kept constant and equal to its initial value during classical control. The mathematical relation used is given in Eq. 22.

22 $$\begin{aligned} \frac{d \Delta u_v}{dt} = K_p \dot{e}(t) + K_i e(t) \end{aligned}$$

## Results

This work has looked at four cases of operation in VSHP where the first two cases implement both classical and optimal control in VSHP for same loading pattern,the third deals with unanticipated disturbances using PID and MPC and the fourth case looks at the performance of deterministic MPC for overloading. Table 2 gives the overview of all the cases studied in this paper. The cases studied are discussed further in detail individually in the following sections.

**Table 2:** Brief overview of the simulated cases.

Case I	VSHP operation for Step and Ramp changes in load: Ramp change handled better while both PID and MPC struggle a little during step load change MPC stabilizes the system faster while PID results in frequency under and overshoots
Case II	VSHP operation showing reaction to fast load changes: Simulated for a short time to see quick frequency response MPC outperforms the PID in terms of frequency stabilization
Case III	VSHP operation with an unanticipated disturbance : MPC maintains the frequency close to its setpoint but has small oscillations, and PID results in overshoot Tradeoff between small oscillations and overshoot for stability MPC handles disturbances better because of its predictive behavior
Case IV	VSHP operation with a step change in load which overloads the generator: Saturated operation MPC tried to maintain power generation for a few seconds even after the application of overload

### Case I: VSHP operation for step and ramp changes in load

**Fig. 2:**
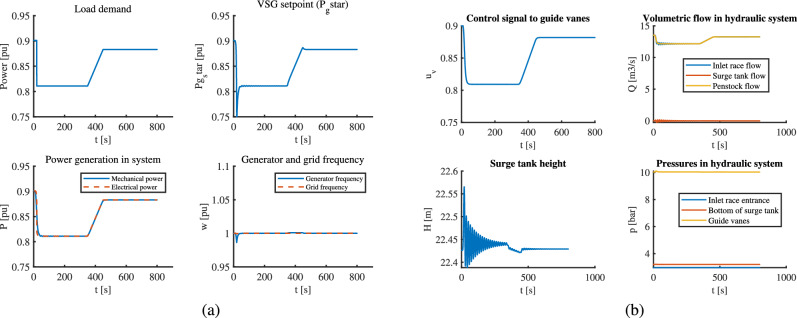
VSHP operation with MPC for step and ramp loading: (**a**) Electric part (**b**) Hydraulic part.

In this case, the VSHP system is subjected to step-down and ramp loads that occur at t = 20 and t = 350 s, respectively. The ramp-up lasts for 100 seconds. Looking at part (a) in Fig. 2 , the MPC makes the reference power to VSG follow exactly the load change, which in turn leads to an almost smooth generator frequency to ramp load. However, for a step change, the reference power undergoes an undershoot that leads to a momentary spike in frequency. This generator frequency under-spike means that the turbine generator unit undergoes vibration during step load change which might cause mechanical damage to the equipment. Again, in part (b) of Fig. 2, we clearly see that MPC prepares the control signal to the guide vanes to make mechanical power follow the load changes due to its feedforward action. The oscillations in upstream flow and surge tank heights are slightly higher for steps than for ramp changes, but both lie within operational limits. The change in pressures in the upstream channels also seems smooth.

**Fig. 3:**
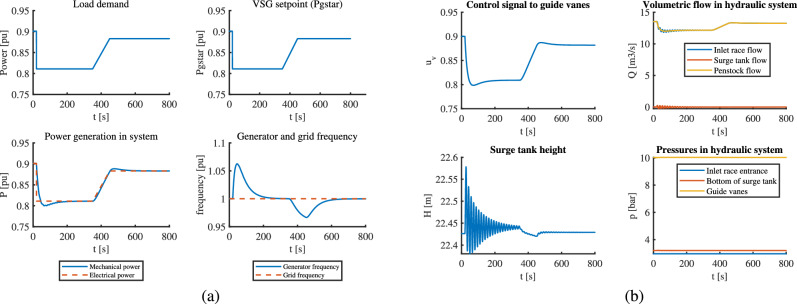
VSHP operation with classical control for step and ramp loading: (**a**) Electric part (**b**) Hydraulic part.

Similarly, the results with classical control for the same loading conditions can be seen in Fig. 3 . The upstream dynamics for flow and pressures look acceptable although larger oscillations are seen, but the control signal to the gates cannot prepare itself to change beforehand by virtue of predictive action as in MPC.This leads to slight mismatch between mechanical and electrical power which further causes frequency variations. This variation is more prominent in step change than in ramp change. However, the controller manages to mitigate frequency variations after some time by virtue of feedback action and runs the system in synchronism.

From these cases, although we see that the MPC clearly outperforms the classical controller, both controllers struggle a little during the step load change. With the MPC, the grid and generator frequencies are brought back to 1 pu much faster than with classical control scheme. In other words, MPC stabilizes the system more quickly. The gradual ramp change is better handled if we see the performance of individual controllers. For step load changes, step size also matters to the controller. A large step size might lead to larger frequency spikes and a violation of constraints.

### Case II: VSHP operation showing reaction to fast load changes

A fast changing load pattern, by preparing a curve that changes for 24 time steps is used to simulate this case. The VSHP system is required to generate power according to the varying load pattern. Classical and optimal control approach is compared from the results. Since the load is changing continuously per second, the simulation time for this case is kept very short. However, this simulation time is enough to compare the performance of two controllers.

**Fig. 4:**
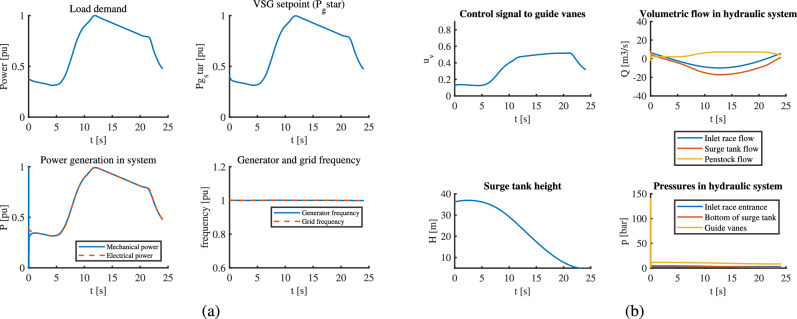
VSHP operation with MPC for a loading pattern that changes continuously: (**a**) Electric part (**b**) Hydraulic part.

In optimal control, the control signal from MPC which is VSG setpoint follows the load pattern exactly, which in turn maintains the generated power and the generator frequency to desired pattern and values as seen in part (a) of Fig. 4. Also, the waterway oscillations are acceptable, and the control signal to guide vanes seems to change smoothly as seen in part (b) of Fig. 4. This is the most realistic case of loading for any hydropower plant, and MPC works well for VSHP with this loading pattern.

**Fig. 5:**
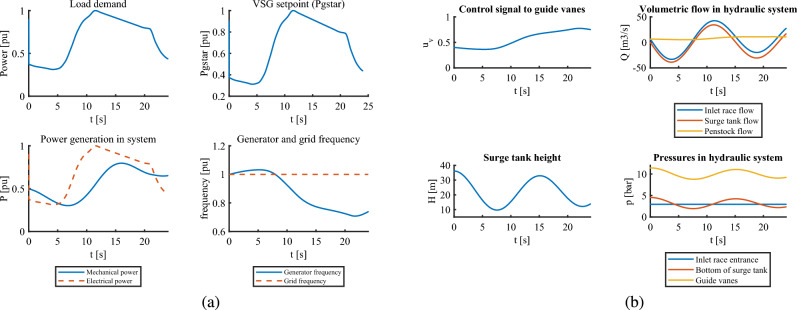
VSHP operation with classical control for a loading pattern that changes continuously: (**a**) Electric part (**b**) Hydraulic part.

When it comes to classical control, the fast changing load pattern is given directly as $P_g^*$ to the VSHP system. The VSHP system is controlled using a PID controller. The results in Fig. 5 show that the PID struggles to change the control signal to guide vanes instantaneously, which leads to mismatch between the mechanical and electrical power due to continuous fast-changing demand pattern. This gives rise to unstable generator frequency and also oscillations in the upstream.This might have occurred because of governor time constant which imposes a delay in PID action. However, had the load change occurred slightly slower, PID might have been able to change to control the signals more effectively. The response-time to change is limited in classical control.

### Case III: VSHP operation with an unanticipated disturbance

In this case, with an initial loading of 0.9034 pu, which is constant throughout the simulation, a sudden disturbance is applied at a simulation time of t = 500 s. This disturbance resembles a sudden unplanned power imbalance experienced by the power plant, and the prediction model used with MPC cannot anticipate this upcoming load disturbance beforehand. For this test, the disturbance is a step function similar to the 10% power imbalance applied to the plant suddenly in an unplanned manner such that the MPC prediction horizon cannot see this coming. Also, the same simulation step is tried with PID as well.

**Fig. 6:**
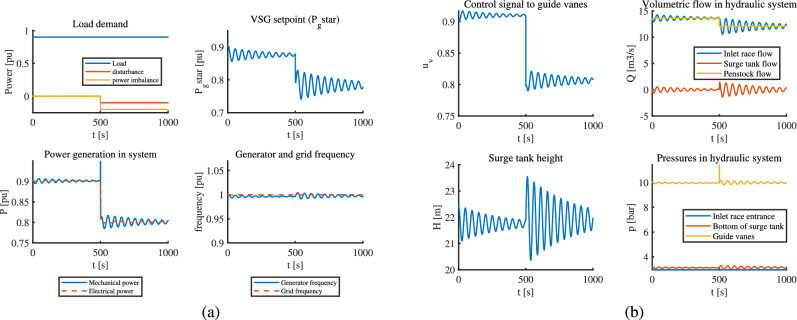
VSHP operation with MPC addressing an unanticipated disturbance: (**a**) Electric part (**b**) Hydraulic part.

The results for MPC show that the generator frequency is kept very close to its reference value and is almost perfect frequency graph, as shown in part (a) of Fig. 6. The control signals $P_g^*$ and $u_v$ undergo minor oscillations which lead to oscillations from upstream to the generated power, however, they lie under the desired limit as seen in part (b) of Fig. 6.

**Fig. 7:**
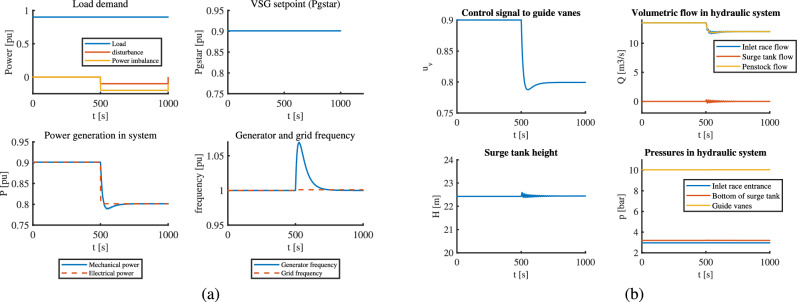
VSHP operation with classical control addressing an unanticipated disturbance: (**a**) Electric part (**b**) Hydraulic part.

Here, the predictive model in MPC cannot see the disturbance coming before it actually happens, and hence only the feedback action of MPC works to meet the objective. This might be the reason for minor oscillations even after rigorous tuning of the MPC. Similarly, the classical control also meets the frequency requirements but the spike of 1.06 pu as seen in part (a) of Fig. 7 during the transient is unacceptable since it lies outside acceptable range of 2.5% allowable frequency deviation according to the standards^3^. The classical controller takes some time to bring the frequency to reference value. Since PID is able to manipulate only one control signal as seen in part (b) of Fig. 7$u_v$, the mechanical power generated changes as per change in guide vanes, and the electrical power generated follows the mechanical power as seen in part (a) of the same figure. This way, the VSG setpoint is untouched. But, above all this, the oscillations in the system seem smaller as compared to MPC. The key takeaway from this case appears to be a trade-off between unacceptable spike on frequency ( with PID) with acceptable oscillations in the system ( with MPC). Of course, MPC looks more viable from stability point of view.

### Case IV: VSHP operation with a step change in load which overloads the generator

**Fig. 8:**
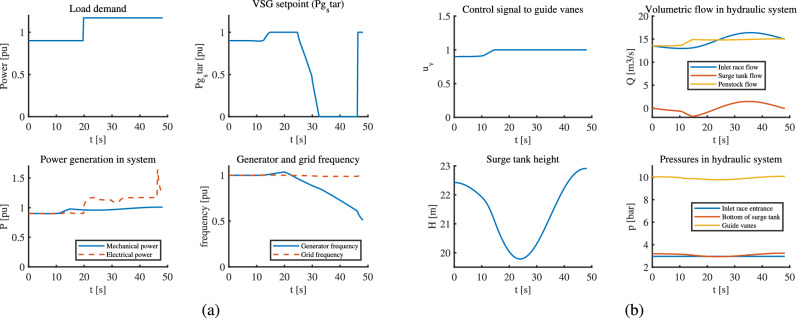
VSHP with MPC undergoing overloaded demand: (**a**) The controller fails to maintain desired frequency due to unaddressed power imbalance (**b**) Changes in hydraulic parameters.

With an initial loading of 0.9034 pu, a 30% load increment is applied at t=20 seconds in this case. The VSHP is clearly overloaded. The MPC changes the VSG setpoint $P_g^*$ before t= 20 seconds by its predictive action to prepare for the coming overload, which makes reference for the electrical power generated as shown in part (a) of Fig. 8, i.e. $P_g^*$ is saturated to its upper limit together with the saturation of the guide vane opening (part (b) of Fig. 8).But after sometime, since the overload cannot be handled by the VSHP for a long time, the MPC fails to generate the required control signal i.e. VSG setpoint. Again, by virtue of predictive behavior MPC prepares for increment in load by starting to act on control signal to guide vanes before t = 20 as seen in same figure. Simultaneously, the frequency also starts to rise before t = 20 to balance for load increment leading to frequency drop coming in future. This case is simulated only for 50 s because the system fails after this time.

The VSHP is successfully supplying the overloaded demand at the cost of short term generator-frequency deviation. This can be seen from the graph of mechanical and electrical power in part (a) of Fig. 8, where the electrical power generated ($P_g$) goes higher than mechanical power for a while, which is maintained by the DC link capacitor. The capacitor is situated between generator side and grid side converter which undergoes heavy discharging during this time. More details about the capacitor in VSHP can be found work by Reigstad et al.^7^. However, the maximum mechanical power is limited to 1 pu which leads to prolonged mismatch between mechanical and electrical power causing the generator frequency to drop and eventually generator failure. Hence, the VSHP cannot be operated with frequency deviation for longer time, however it can undergo allowable frequency deviation during transients depending on the inertia of VSHP and size of capacitor. The upstream dynamics seems fine even during overload because of the constraint in gate opening which cannot go above 100%. However, PID is not used for this case since it cannot manipulate the VSG setpoint and has no scope for improvement for addressing this case unlike MPC.

### Superiority of MPC over PID

**Fig. 9:**
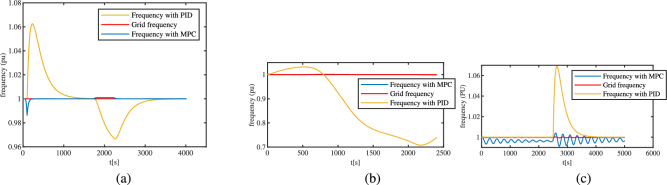
Comparison of PID and MPC based on maintaining generator frequency to desired setpoint (grid frequency) for: (**a**) Step and ramp loading, (**b**) Fast changing load (**c**) Unanticipated disturbance occurring in a constantly loaded VSHP at half time.

In Variable Speed Hydropower control, the main objective is to maintain the minimum deviation in turbine-generator unit speed, while supplying the quickly changing power demand. The generator frequency with PID and MPC for the loading pattern in case I, II and III is compared in three sub-figures of Fig. 9. The figures show a higher frequency deviation peaks up to an unacceptable value when the hydropower is controlled by PID for all three cases studied. The frequency, in the case of classical control, takes some time after the change in load to come back to its setpoint in case of step and ramp loading as seen in sub-figure (a) of Fig. 9, but the controller fails to maintain a stable frequency in the case of continuously changing load as seen in sub-figure (b) of Fig. 9. This is due to slower feedback action of the classical controller because of large time constant of mechanical system due to inertia. However, the predictive action of MPC enables it to address the changing load more smoothly (the blue line overlaps the red). Similarly, when an unanticipated disturbance is given at half of the simulation time, the frequency controlled by PID reaches an unacceptable peak and settles down, however the frequency controlled by MPC does not violate the frequency limits but undergoes some oscillations as seen in sub-figure (c) of Fig. 9. Since the predictive model fails to see the unanticipated disturbance apriori, only the feedback action works to address the disturbance here. Furthermore, performance metrics comparing of NLMPC and classical PID for time-response of frequency are listed for the three cases compared in Table 3. Based on Integral of Squared Error (ISE), Integral of Absolute Error (IAE), settling time and percentage overshoot, we can clearly claim that NLMPC outperforms the classical PID.

**Table 3:** Quantative comparison of MPC and PID controllers under different operating cases.

Metrices	Case I		Case II		Case III	
	MPC	PID	MPC	PID	MPC	PID
ISE	0.001009976	0.295002602	$7.76\times 10^{-6}$	0.789891356	$1.47\times 10^{-2}$	0.251183068
IAE	0.228539368	8.964718725	0.00887965	3.449963113	3.380125539	5.586567084
Settling Time (s)	$4.53\times 10^{-12}$	486.6	$7.246\times 10^{-12}$	22.99	$9.4352\times 10^{-12}$	602.2
Overshoot (%)	0.0995	6.2542	0.3750	3.1876	0.4072	6.9166

For the power system stability, frequency limits violation is a serious issue. For a VSHP, although a momentary frequency violation is allowable, but the control system should try to bring it back to the synchronous value as soon as possible to prevent further slowing down of the turbine which would then be difficult to bring back to synchronous speed. This momentary deviation is supposed to be handled by the kinetic evergy stored in the turbine, and the dc-link capacitor on the converters unit. Therefore, the magnitude and time for allowable frequency deviation in a VSHP depends on the moment of inertia of its turbine and the size of capacitor.

With the increase in the complexity of the grid by the injection of intermittent renewable sources and dynamic loads, the load demand varies very quickly in modern grids. This requires the control system to be more demanding to maintain stability. MPC as seen to address the continuous changing demand very smoothly, and working well even with the disturbance, can be a very good alternative to the existing classical controllers. With the requirement of well-trained manpower for the operation of VSHP, the computationally intensive MPC would not be a big hurdle to implement. Furthermore, a brief summary of comparison derived from the three cases studied can be seen in Table 4. Though predictive controllers are a little demanding in case of model accuracy, parameter sensitivity and computational complexity, they clearly outperform the classical controllers and ensure the stable operation of both grid side and upstream side through tighter control and constraint handling ability.

**Table 4:** Comparison of classical controllers and MPC performance in the control of VSHP.

Aspect	Classical control	NLMPC
Power balance	Larger power imbalances	Smaller deviations during transition Predictive behavior for load changes
Hydraulic system	Larger waterway oscillations Less smooth guide vanes operation	Faster wave damping Smoother guide vane operation
Generator control	Struggles with frequency during load changes Single control input limits performance	Tight frequency control Adapts to changing conditions Better handling of continuous changes
Response to changes	Reactive control Limited by fixed PI parameters	Anticipatory behavior Adapts to changing conditions Handles multiple continuous changes
Limitations	Fixed control structure Limited coordination capability	Computationally intensive Requires accurate model Parameter sensitivity

## Discussion

The biggest benefit of MPC implemented here is that it successfully coordinated hydraulic and electric system control. This coordinated control comes with consideration of upstream water oscillation wave-cycle time and electric time constant together. The prediction horizon length needed to be at least one wave cycle long for the model to be able to predict complete behavior of system during oscillation. This horizon worked well for electric system since it is faster than the hydraulic system.The length of prediction horizon directly impacts the computational speed of MPC. Too short prediction horizon leads to myopic control though the computational speed is faster, and too long horizon length heavily increases the computational burden. Furthermore, tuning the weighting matrices in objective function also involves understanding of the system. Increasing Q led to faster setpoint tracking but slower control action, however decreasing H led to aggressive control. Faster control is desired for electrical control (VSG setpoint), however the system experiences water hammer if the guide vanes are operated too fast. Also, minimizing the frequency error fast enough is another priority as this has impact on system stability. So, the H and Q matrix were relatively tuned for getting optimal control. Also, while dealing with multi-objective control, this scheme gives us the flexibility to prioritize the desired action. Here frequency balance is taken as first priority.

In addition to this, computation time for MPC is another key factor in its implementation. This gives information about how long the controller takes to generate a control input to minimize the error. In this paper, different cases are simulated for different time lengths depending on the nature of loading. While the total computation time depends on this pre-decided simulation length, the computation time for each timestep, is the time taken by the optimizer to solve the optimization problem once at every sampling time. The average computation time gives better insights on how fast the MPC worked for each cases. Table 5 states average computation time for simulation for each case.

**Table 5:** Average computation time per loop for different operating cases.

Cases	Average computation time per simulation (s)
Case I	5.498653
Case II	8.291017
Case III	1.544326
Case IV	2.067105

With the modern grid going towards more renewable sources (power electronic converters), concept of isolated or grid-connected microgrids emerging, non-linear loads in the system, etc, the grid is very likely to undergo sudden unanticipated disturbances. The disturbances may be of any kind like sudden load loss, harmonics injection, sudden generation source failure leading to overload for other generators, natural calamities, contingency, adverse weather conditions, etc. This unexpected event might sometimes include intermittent supply by renewable sources like wind, Photovoltaic, etc which are weather-dependent. Modern control strategies for any generation source need to prioritize these unanticipated events for power grid support. This work tries to touch this area of uncertainties by testing the controllers (MPC and PID) for a sudden disturbance in VSHP in case III. Although the type of disturbance used here is very generalized in the form of a step load imbalance, the cause of this imbalance might root to the above mentioned reasons. Trying to look at the control performance of classical control and MPC for the disturbance-injected system, the case offers a tradeoff between frequency spike and system oscillations. But, the MPC although giving acceptable results, has room for improvement by incorporating state and/or parameter estimation and even going for stochastic MPC. More research is required to address these unexpected events.

The load demand for any power plant might not always lie under its capability to supply. Sometimes it might have to undergo overloading.The VSHP can withstand overload for a while since it can deviate away from synchronous operating speed for sometime. The time to withstand the overloading depends on its size and the amount of overload. This capability of VSHP would be very beneficial during the transient operation. However, prolonged operation in overloaded condition might decay the frequency to unacceptable value and the VSHP might eventually stop as seen in case IV. To address this problem, the controllers must be designed to share the load to other generators existing in the grid by controlling the VSHP to give maximum power it can. However, the other generators might take some time to ramp-up their generation to meet additional load and VSHP can be designed to handle the overload during this ramp-up by preventing the grid frequency decay. This can be achieved using this multi-objective MPC by including a grid with multiple sources in this work. More research with a grid model representing clear grid dynamics would be another way forward to grid support using VSHP controlled by NLMPC.

## Conclusion and future work

After having tested the Variable Speed Hydropower Plant with various loading patterns and perturbation conditions under classical and predictive control approaches, we conclude that Model Predictive Control is a promising control solution for VSHP. This conclusion is further backed up by the comparison with classical control approach based on time-domain plots and performance metrics. Small computation time (in s) makes it implementable in realistic scenarios to control the variable speed power plant since VSHPs have some allowed frequency deviation and the few seconds computation time is enough for the control algorithm to work. However, its successful implementation requires an adequate system modeling and control design. VSHPs themselves are highly complicated systems involving the interaction of hydraulic dynamics, rotational mechanics, power electronic converters, and grid response. The ability of proposed NLMPC to optimize multi-objectives and adhere to system constraints makes it suitable for such integrated systems. In addition, ability of VSHP to offer flexible operation is further enhanced by the proposed control method, which enables coordinated control of the hydraulic and electrical subsystems.

For the case of contemporary power grids, which experience frequent disturbances and blackouts because of high penetration of renewable sources and low system inertia, VSHP would provide tremendous grid support potential. With the optimal control implemented in this paper, the VSHP quickly changes its power output through converter-based control systems to mimic synthetic inertia and supply primary frequency response. This capability is required to arrest frequency nadirs and support the Rate of Change of Frequency, thus enabling the VSHP to become effective participant in frequency regulation and reserve ancillary service markets. This guides us to an interesting research direction: study of optimally controlled VSHPs’ contribution to energy markets, which would highlight the potential economic and financial benefits it can offer. Also, an important but not fully investigated feature is VSHPs’ reactive power and voltage support capability. Additional research in this direction can further increase their capability to increase grid stability, especially as power systems evolve to low-inertia, renewable-based systems. Furthermore, the future direction also can expand to experimental implementation of this work which could contribute in further improvising this control strategy and validation with real-time models.

## Data Availability

The data supporting the findings of this study on Non-linear Model Predictive Control for Variable Speed Hydropower are available upon request from the corresponding author. The data includes the specifications and sizes for each component of variable speed hydropower plant used, including the details of upstream waterways. This data and details of the model is also published and publicly accessible in our previous work (DOI: 10.4173/mic.2025.1.1). No proprietary or third party data were used in this study. The computational models and scripts developed for simulation can also be shared upon request for academic and research purposes.

## Abbreviations

$A_i$: Cross-sectional area of inlet pipe
$L_i$: Length of inlet pipe
$P_{R2}$: Pressure at intake point of reservoir
$P_{s,1}$: Pressure at the bottom of surge tank
$\rho$: Density of water
*g*: Acceleration due to gravity
$D_i$: Diameter of inlet race pipe
*f*: Coefficient of friction
$Q_i$: Flow in inlet pipe
$A_i$: Surface area of inlet race pipe
$A_{i,w}$: Wetted area of inlet race
$H_i$: Vertical height of inlet pipe (height difference between start and end point)
$Q_s$: Flow in surge tank
$Q_P$: Flow in penstock
$H_s$: Water level in surge tank
$A_s$: Surface area of surge tank
$A_P$: Cross-sectional area of penstock
$P_{s,1}$: Pressure at the bottom of surge tank
$$P_a$$: Atmospheric pressure
$L_P$: Length of penstock
$D_P$: Diameter of penstock
$H_p$: Vertical height of penstock
$P_t$: Pressure in turbine
$Q_P$: Flow in penstock
$C_v$: Turbine valve capacity (nominal information of hydro-turbine system)
$u_v$: Control signal to guide vanes
$\omega$: Speed of turbine-generator unit
$$R_1$$: Inlet radius
$$A_1$$: Inlet area
$$R_2$$: Outlet radius
$$A_2$$: Outlet area
$P_{\text {shaft}}$: Mechanical power output from turbine (MW)
$\alpha _1$: Guide vane opening
$$\beta _2$$: Turbine constant
$P_{\text {base}}$: Base value of mechanical power (MW)
$P_m$: Mechanical power input to the generator (pu)
$P_g$: Electrical power injected by VSHP to the grid (pu)
*H*: Moment of inertia (pu)
$\omega _{\text {gen}}$: Frequency of turbine-generator unit (pu)
$\Delta \omega _{\text {gen}}$: Change in frequency of generator (pu)
*D*: Damping coefficient (pu)
$\omega _{\text {grid}}$: Grid frequency (pu)
$\omega _{\text {grid}}$: Grid frequency (pu)
$\omega _{\text {ref}}$: Reference frequency for the grid (pu)
$P_{\text {gen}}$: Total active power generation in the grid (pu)
$P_{\text {load}}$: Total active power demanded by the grid (pu)
$P_{\text {dist}}$: Disturbances or power imbalances in the grid (pu)
$D_m$: Damping coefficient for the grid
$H_g$: Mean grid inertia (pu)
$S_n$: Total rated power of all connected sources (pu)

## References

[1] (IEA), I. E. A. Electricity mid-year update - july 2024 (2024). Licence: CC BY 4.0.

[2] Key, T., Rogers, L., Brooks, D. & Tuohy, A. Quantifying the value of hydropower in the electric grid: Final report . Final project report on hydropower valuation in the electric grid 10.2172/1057586 (2012).

[3] Acosta, M. N., Pettersen, D., Gonzalez-Longatt, F., Peredo Argos, J. & Andrade, M. A. Optimal frequency support of variable-speed hydropower plants at Telemark and Yestfold, Norway: Future scenarios of nordic power system. *Energies* **13**, 3377. 10.3390/en13133377 (2020).

[4] Fraile-Ardanuy, J., Wilhelmi, J., Fraile-Mora, J. & Perez, J. Variable-speed hydro generation: Operational aspects and control. *IEEE Trans. Energy Conversion* **21**, 569–574. 10.1109/TEC.2005.858084 (2006).

[5] Reigstad, T. I. & Uhlen, K. Virtual inertia implementation in variable speed hydropower plant. In *2019 Modern Electric Power Systems (MEPS)* 1–6 (2019). 10.1109/MEPS46793.2019.9395013.

[6] WindEurope. Iberian peninsula blackout proves the need for grid resilience. WindEurope Newsroom (2025). Online accessed June 2025.

[7] Reigstad, T. & Uhlen, K. Modelling of variable speed hydropower for grid integration studies. *IFAC-PapersOnLine* **53**, 13048–13055. 10.1016/j.ifacol.2020.12.2176 (2020) (**21st IFAC World Congress**).

[8] Lan, Z. *et al.* Benefits of variable speed pumped hydro storage technology for increasing renewable integration in regional power grids. In *2021 IEEE 5th Conference on Energy Internet and Energy System Integration (EI2)*, 660–664, (2021). 10.1109/EI252483.2021.9712853.

[9] Wu, P. *et al.* Impact of turbine governor on stability of variable-speed pumped storage units with full-size converter. In *2023 IEEE 7th Conference on Energy Internet and Energy System Integration (EI2)*, 4116–4122, (2023). 10.1109/EI259745.2023.10512565.

[10] Kumari, R., Prabhakaran, K. K. & Chelliah, T. R. Enhanced control of large rated variable speed hydrogenerating unit under unbalanced grid voltage conditions. In *2021 IEEE International Conference on Environment and Electrical Engineering and 2021 IEEE Industrial and Commercial Power Systems Europe (EEEIC / I & CPS Europe)*, 548–553, (2021). 10.1109/EEEIC/ICPSEurope51590.2021.9584755.

[11] Belhadji, L., Bacha, S. & Roye, D. Direct power control of variable-speed micro-hydropower plant based on propeller turbine. In *2012 XXth International Conference on Electrical Machines*, 2079–2084, (2012). 10.1109/ICElMach.2012.6351843.

[12] Zhou, Q., Luo, D., Dai, L., Wu, B. & Luo, H. Mppt control strategy of variable speed hydropower system based on improved fuzzy control. In *2021 IEEE 4th Student Conference on Electric Machines and Systems (SCEMS)*, 1–6, (2021). 10.1109/SCEMS52239.2021.9646103.

[13] Beus, M. & Pandžić, H. Application of model predictive control algorithm on a hydro turbine governor control. In *2018 Power Systems Computation Conference (PSCC)*, 1–7, (2018). 10.23919/PSCC.2018.8442594.

[14] Beus, M. & Pandžić, H. Practical implementation of a hydro power unit active power regulation based on an mpc algorithm. *IEEE Trans. Energy Conversion* **37**, 243–253. 10.1109/TEC.2021.3094059 (2022).

[15] Nagode, K., Škrjanc, I. & Murovec, B. Enhanced stability and failure avoidance of hydropower plant in contingent island operation by model predictive frequency control. *Energy Rep.* **8**, 9308–9330. 10.1016/j.egyr.2022.07.040 (2022).

[16] Yadav, G. Enhancing power quality and stability in grid-interactive electric vehicle chargers using advanced control strategies and disturbance compensation. *Eng. Res. Express* **7**, 035321. 10.1088/2631-8695/adf038 (2025).

[17] Pandey, M., Winkler, D., Sharma, R. & Lie, B. Using mpc to balance intermittent wind and solar power with hydro power in microgrids. *Energies* **14**, 874. 10.3390/en14040874 (2021).

[18] Ye, S. et al. Real-time model predictive control study of run-of-river hydropower plants with data-driven and physics-based coupled model. *J. Hydrol.* **617**, 128942. 10.1016/j.jhydrol.2022.128942 (2023).

[19] Gao, J. et al. Variable-speed hydropower generation: System modeling, optimal control, and experimental validation. *IEEE Trans. Ind. Electron.* **68**, 10902–10912. 10.1109/TIE.2020.3031528 (2021).

[20] Reigstad, T. I. & Uhlen, K. Variable speed hydropower conversion and control. *IEEE Trans. Energy Conversion* **35**, 386–393. 10.1109/TEC.2019.2943233 (2020).

[21] Reigstad, T. I. & Uhlen, K. Optimized control of variable speed hydropower for provision of fast frequency reserves. *Electr. Power Syst. Res.* **189**, 106668. 10.1016/j.epsr.2020.106668 (2020).

[22] Reigstad, T. I. & Uhlen, K. Nonlinear model predictive control of variable speed hydropower for provision of fast frequency reserves. *Electr. Power Syst. Res.* **194**, 107067. 10.1016/j.epsr.2021.107067 (2021).

[23] Vasudevan, K. R., Ramachandaramurthy, V. K., Venugopal, G., Ekanayake, J. B. & Tiong, S. K. Variable speed pumped hydro storage: A review of converters, controls and energy management strategies. *Renew. Sustain. Energy Rev.* **135**, 110156 (2021).

[24] Xu, Z., Deng, C. & Yang, Q. A primary frequency control strategy for variable-speed pumped-storage plant in generating mode based on adaptive model predictive control. *Electr. Power Syst. Res.* **221**, 109356. 10.1016/j.epsr.2023.109356 (2023).

[25] Nepal, T., Øyvang, T., Bista, D. & Sharma, R. Dynamic model for control of variable speed hydropower plant. *Modeling Identification Control* **46**, 1–11. 10.4173/mic.2025.1.1 (2025).

[26] Lie, B. *Lecture notes in modeling of dynamic systems* (University of South Eastern Nrway, 2019).

